# Draft Genome Sequence of Strain LSUCC0057, a Member of the SAR92 Clade of *Gammaproteobacteria*

**DOI:** 10.1128/MRA.00599-19

**Published:** 2019-06-20

**Authors:** V. Celeste Lanclos, Michael W. Henson, Chase Doiron, J. Cameron Thrash

**Affiliations:** a Department of Biological Sciences, University of Southern California, Los Angeles, California, USA; b Department of Biological Sciences, Louisiana State University, Baton Rouge, Louisiana, USA; Georgia Institute of Technology

## Abstract

We present the draft genome sequence of strain LSUCC0057, a member of the SAR92 clade of *Gammaproteobacteria*, isolated from coastal waters near Buras, LA. The genome contains proteorhodopsin and indicates the potential for aerobic heterotrophy, assimilatory sulfate reduction, and carotenoid biosynthesis.

## ANNOUNCEMENT

The SAR92 clade belongs to the oligotrophic marine *Gammaproteobacteria* (OMG) (1) in the family *Porticoccaceae*, order *Cellvibrionales* (2). These organisms occur in high abundance in coastal environments (2) and may benefit from the use of alga-derived by-products for their growth (3). The first isolated strain, HTCC2207, was obtained from the Oregon coast, and its genome also indicated proton-pumping proteorhodopsin and carotenoid synthesis genes (4). We isolated LSUCC0057 from the coast of Buras, LA, using high-throughput dilution-to-extinction cultivation with an artificial seawater medium (5). Phylogenetic placement using the 16S rRNA gene (5) grouped LSUCC0057 with other known SAR92 isolates (4). However, to our knowledge, LSUCC0057 is the first SAR92 isolate from the Gulf of Mexico. We therefore sequenced its genome to provide opportunities for comparative genomics and biogeography.

Cultures from four replicate LSUCC0057 cryostocks originating from the same inoculum were grown in JW1 medium at room temperature (5), and DNA was extracted and combined as previously reported (6). Library preparation and sequencing were completed at the Argonne National Laboratory Environmental Sample Preparation and Sequencing Facility. DNA was sheared with a Covaris sonicator (Woburn, MA), and library prep was done using the PrepX ILMN library kit on the Apollo324 system (Mountain View, CA), following the manufacturer’s instructions. Libraries were size selected with the Sage Science BluePippin system (Beverly, MA), and DNA was sequenced using an Illumina MiSeq instrument. We screened and assembled 650,694 2 × 251-bp reads with the A5 MiSeq pipeline, using default settings (7). The final assembly was a 2,203,110-bp genome in 13 contigs, with an *N*_50_ value of 1,125,525 bp and a GC content of 59.3%. The median coverage was 66×. The genome was estimated to be 99.26% complete, with 92.8% coding density and 0.37% contamination determined via CheckM, with default settings (8). Annotation through the NCBI Prokaryotic Genome Annotation Pipeline (PGAP) (9) predicted 2,017 total genes, comprising 1,958 protein-coding, 40 tRNA, and 9 rRNA genes (3 copies each of the 5S, 16S, and 23S genes), 4 other RNA genes, and 6 pseudogenes.

Preliminary metabolic reconstruction with GhostKOALA (10) indicated that the LSUCC0057 genome contains genes for aerobic heterotrophy, including both high- and low-affinity cytochrome *c* oxidases (11), and the following complete pathways: glycolysis, gluconeogenesis, the tricarboxylic acid (TCA) and glyoxylate cycles, the pentose phosphate pathway, one carbon pool by folate, steroid degradation, and assimilatory sulfate reduction. It contains ABC transporters for zinc (*znuABC*), phosphate (*ptsSCAB*), lipoprotein (*lolCED*), and lipopolysaccharide (*lptFGB*); two-component systems for response to nitrogen (*glnLG*) and phosphate (*phoRB*); and a putative proteorhodopsin gene (78.6% amino acid identity shared with that in HTCC2207 via blastp) with upstream carotenoid production genes (*crtBYl*) like those in HTCC2207 (4).

**FIG 1:**
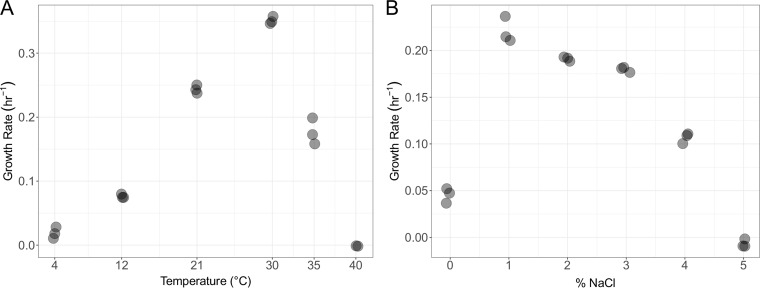
Growth rates of LSUCC0057 at various temperatures (A) and salinities (reported as % NaCl) (B).

We tested the growth of LSUCC0057 across different temperatures and salinities. Temperature experiments used JW1 medium (5). Salinity experiments were completed at 24°C by adding various percentages of NaCl while maintaining all other JW1 components. Experiments were conducted in triplicate, and growth rates were calculated as reported previously (12). LSUCC0057 grew at temperatures of 4 to 35°C (optimum, 30°C) and salinities of 0 to 4% NaCl (optimum, 1%) (Fig. 1).

### Data availability.

The genome sequence is available in GenBank under the BioProject number PRJNA528533; the Illumina reads are available in the SRA under accession number SRR8890679. Cryostocks and/or live cultures of LSUCC0057 are available upon request.
